# Cellular Responses of the Lichen *Circinaria gyrosa* in Mars-Like Conditions

**DOI:** 10.3389/fmicb.2018.00308

**Published:** 2018-03-05

**Authors:** Rosa de la Torre Noetzel, Ana Z. Miller, José M. de la Rosa, Claudia Pacelli, Silvano Onofri, Leopoldo García Sancho, Beatriz Cubero, Andreas Lorek, David Wolter, Jean P. de Vera

**Affiliations:** ^1^Departamento de Observación de la Tierra, Instituto Nacional de Técnica Aeroespacial, Madrid, Spain; ^2^Instituto de Recursos Naturales y Agrobiología de Sevilla, Consejo Superior de Investigaciones Científicas, Sevilla, Spain; ^3^Department of Ecological and Biological Sciences, University of Tuscia, Viterbo, Italy; ^4^Departamento de Biología Vegetal II, Universidad Complutense, Madrid, Spain; ^5^German Aerospace Center (DLR) Berlin, Institute of Planetary Research, Berlin, Germany

**Keywords:** Mars environment, extremotolerance, lichens, *Circinaria gyrosa*, photosynthetic activity, analytical pyrolysis

## Abstract

Lichens are extremely resistant organisms that colonize harsh climatic areas, some of them defined as “Mars-analog sites.” There still remain many unsolved questions as to how lichens survive under such extreme conditions. Several studies have been performed to test the resistance of various lichen species under space and in simulated Mars-like conditions. The results led to the proposal that *Circinaria gyrosa* (Lecanoromycetes, Ascomycota) is one of the most durable astrobiological model lichens. However, although *C*. *gyrosa* has been exposed to Mars-like environmental conditions while in a latent state, it has not been exposed in its physiologically active mode. We hypothesize that the astrobiological test system “*Circinaria gyrosa*,” could be able to be physiologically active and to survive under Mars-like conditions in a simulation chamber, based on previous studies performed at dessicated-dormant stage under simulated Mars-like conditions, that showed a complete recover of the PSII activity (Sánchez et al., 2012). Epifluorescence and confocal laser scanning microscopy (CLSM) showed that living algal cells were more abundant in samples exposed to niche conditions, which simulated the conditions in micro-fissures and micro-caves close to the surface that have limited scattered or time-dependent light exposure, than in samples exposed to full UV radiation. The medulla was not structurally affected, suggesting that the niche exposure conditions did not disturb the lichen thalli structure and morphology as revealed by field emission scanning electron microscopy (FESEM). In addition, changes in the lichen thalli chemical composition were determined by analytical pyrolysis. The chromatograms resulting from analytical pyrolysis at 500°C revealed that lichen samples exposed to niche conditions and full UV radiation consisted primarily of glycosidic compounds, lipids, and sterols, which are typical constituents of the cell walls. However, specific differences could be detected and used as markers of the UV-induced damage to the lichen membranes. Based on its viability responses after rehydration, our study shows that the test lichen survived the 30-day incubation in the Mars chamber particularly under niche conditions. However, the photobiont was not able to photosynthesize under the Mars-like conditions, which indicates that the surface of Mars is not a habitable place for *C*. *gyrosa*.

## Introduction

Lichens are structurally complex organisms resulting from a symbiotic relationship of algae, also known as cyanobacteria (photobiont), and ascomycetes, also known as basidiomycetes (mycobiont). Some lichens may also be composed of a third partner consisting of a specific basidiomycete yeast, which forms the cortex of the lichen thallus (Spribille et al., 2016). When exposed to environmental stress (such as extreme temperatures, desiccation, and UV radiation), lichens develop photoprotective mechanisms, including light scattering, radiation screening, thermal dissipation, antioxidant defense, membrane repair, and macromolecular production (Nguyen et al., 2013).

Lichens may produce several secondary metabolites as adaptive mechanisms for growing in harsh living conditions. Direct gas chromatography (GC)–mass spectroscopy (MS) analysis of a solvent extract is not straightforward because of non-volatile residues, which remain undetected because they are trapped in the chromatographic system (Stojanovic et al., 2011). Thus, we propose direct analyses of the samples by analytical pyrolysis (Py-GC/MS), which is defined as the thermochemical decomposition of organic materials at elevated temperatures in the absence of oxygen. The pyrolysis products (pyrolysates) are amenable to chromatographic separation, which in combination with an MS detector (GC/MS), provide a fingerprint for the molecular structure, including complex mixtures of macromolecular substances (De la Rosa et al., 2009). Pyrolytic techniques have additional well-known advantages such as the requirement of small sample sizes and minimal, if any, sample preparation. Thus, Py-GC/MS has become an important tool for analytical characterization of a wide spectrum of complex carbonaceous matrices including fossil organic matter, algal and vascular plants, sediments, and urban dust (Lee et al., 2005; De la Rosa et al., 2008; Pereira de Oliveira et al., 2011; Braovaca et al., 2016). This technique has already been successfully used to determine changes in lichens' chemical composition under different environmental conditions (MacGillivray and Helleur, 2001). It is based on the fact that lichens often react to stress factors by changing their acids as the result of a defense- or stress-induced metabolism. Rikkinen (1995) studied lichens' response to UV light. It has been reported that lichens under harmful stress condition will alter their composition, either as a chemical defense or in response to damage (Treshow and Anderson, 1989; Richardson, 1992).

Several studies have been performed to test the response of various lichen species to harsh conditions in space and Mars-like conditions on board space missions. Space is an extremely hostile environment, which is characterized by a high vacuum (10^−7^–10^−4^ Pa), an intense field of ionizing radiation of solar and galactic origin, unfiltered solar ultraviolet (UV) radiation, and extreme temperatures (−120 to +120°C). Among the tested organisms, bacterial spores of *Bacillus subtilis* (Firmicutes, Bacillales) (Horneck et al., 1984, 2001; Horneck, 1993; Rettberg et al., 2002), the lichens *Rhizocarpon geographicum, Xanthoria elegans* (Sancho et al., 2007), and *Circinaria gyrosa* (De la Torre et al., 2010), and adults and eggs of the tardigrades *Richtersius coronifer* and *Milnesium tardigradum* (Jönsson et al., 2008) turned out to be the most resistant to these conditions. About 70% of the *B. subtilis* spores survived 2,107 days in space on board the National Aeronautics and Space Administration Long Duration Exposure Facility after being shielded from solar UV (Horneck et al., 1994). However, direct exposure to the solar extra-terrestrial UV radiation, as experienced in space, reduced their survival by orders of magnitude. So far, lichens are the only organisms that were able to survive under space conditions, which included solar extraterrestrial UV radiation as tested during the 2-week flight of BIOPAN-5 and-6 (Sancho et al., 2007; De los Ríos et al., 2009; De la Torre et al., 2010). Other studies were performed to concurrently test the response of lichen species under space and Mars-simulated conditions on board two space missions, EXPOSE-E and -R2 (Onofri et al., 2012; 2014–2016 mission), on the International Space Station. Laboratory studies using a simulated Mars environment and space-relevant ionizing radiation have also been conducted (Sánchez et al., 2012; De la Torre et al., 2017; Meeßen et al., 2017). The results showed that the species most resistant to space and Mars conditions was the vagrant lichen species *C. gyrosa* in a latent state. *Circinaria gyrosa* (formerly *Aspicilia fruticulosa*, Sohrabi et al., 2013) is a vagrant fruticose lichen with a crustose growth form. It does not grow while attached to a substrate. It is from the steppes and shrub lands with outliers in Southwest Asia and the Mediterranean area of Spain (Sohrabi et al., 2013). This lichen is characterized by a coralloid thallus with dichotomous branching and a compact internal structure (Sancho et al., 2000; Meeßen et al., 2013). Externally, it is limited by a thick cortex that provides efficient shielding against the hostile parameters of outer space (Sancho et al., 2007) and planetary conditions such as those seen on Mars. Yet, physiologically active *C*. *gyrosa* has not been exposed to Mars' environmental conditions. For this reason, our aim was to perform a first-time multidisciplinary assessment of *C*. *gyrosa*'s viability and morphological changes after exposure to the Mars conditions. Lichen viability was assessed by fluorescence microscopy as chlorophyll-induced red autofluorescence is used as an indicator of cell viability such as seen with intact chlorophyll molecules, which display red fluorescence in living cells. This method has been used to assess terrestrial phototrophic microorganisms' and marine phytoplanktons' viability in addition to a plant's ability to tolerate stress factors (Hense et al., 2008; Osticioli et al., 2013) since the red color of photosynthetic cells under UV light (Gausla and Ustvedt, 2003) is related to optimal photosynthetic activity that produces colored light or absence of light in cases of cell damage. Recently, this technique has been introduced in the field of astrobiology for examination of the viability of lichens exposed to simulated space conditions in order to help to distinguish intact cells from cells injured by high-dose ionizing radiation (De la Torre et al., 2017). In addition, changes in the chemical composition of the lichen thalli were determined by analytical pyrolysis (Py-GC/MS).

## Materials and methods

### Sample preparation and irradiation

We chose the lichen species *C. gyrosa* (Sohrabi et al., 2013) for the Mars simulation experiment. It is characterized by a coralloid thallus with dichotomous branching and a compact internal structure (Sancho et al., 2000; Meeßen et al., 2013). All samples were collected from clay soils of the Central Spain steppic highlands, which are characterized by extreme insolation, high temperature contrasts, and arid summers (Crespo and Barreno, 1978). In the presence of these extremely harsh environmental conditions, *C. gyrosa* will enter a desiccated state in the form of a metabolic suspension or stasis (known as cryptobiosis or latent state), in which the cells of the lichen symbionts are dehydrated, and most biochemical activity ceases.

**Figure 1:**
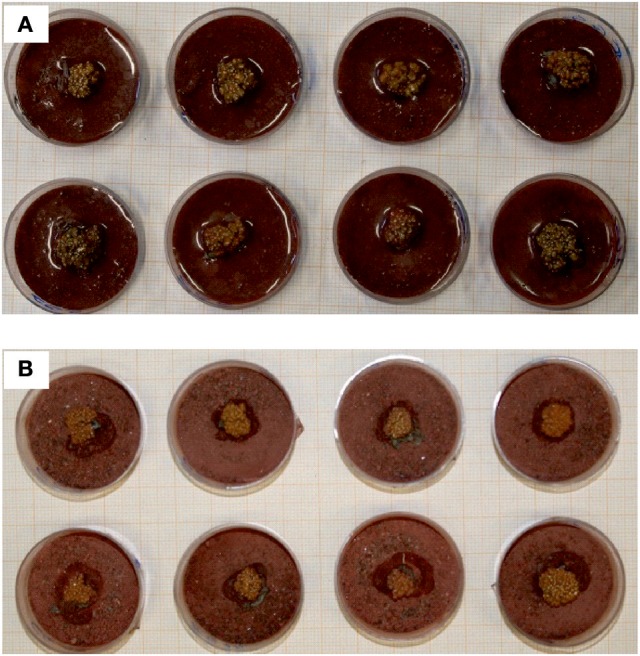
Samples of *Circinaria gyrosa* used in this work. **(A)** Before Mars simulation experiment. **(B)** After Mars simulation experiment.

Replicate samples of *C. gyrosa* with 10 mm diameter were used and classified into two groups: (i) the first was composed of eight exemplars for exposure to simulated Mars environmental conditions (Cg1–Cg8; Figure 1), and (ii) the second, also formed with eight samples, was used as laboratory control (Cg9–Cg16). Samples for the Mars simulation experiment were again classified into two sets, each composed of four samples: (i) the first set was used for niche-led conditions (Cg1–Cg4), and (ii) the second set included samples that were exposed to full UV radiation (Cg5–Cg8). Before the Mars experiment, all samples were activated (as described below), and the photosynthetic activity was measured.

### Description of experiment equipment

**Figure 2:**
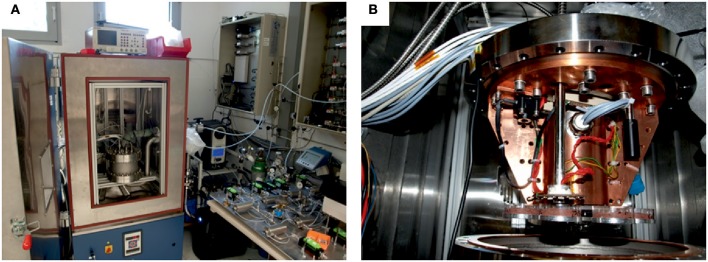
Mars Simulation Facility at DLR. **(A)** Closed experiment chamber. **(B)** Opened experiment at DLR-chamber.

The experiment was carried out at the Mars Simulation Facility (MSF) of the DLR Institute of Planetary Research in Berlin (Lorek and Koncz, 2013, Figure 2A). The main part of the MSF is a climate chamber (CC) with inside dimensions of 80 cm height, 60 cm depth, and 50 cm width. The experiment was performed in an experimental chamber (EC), a vacuum sealed stainless steel chamber with a volume of 10.3 L inside the CC (Figure 2B). The EC has electrical connectors, connectors for gas in and output, four fibers for xenon light, and one fiber for photosynthetic activity measurements obtained with a photosynthesis yield analyzer (MiniPAM, Walz GmbH, Germany) as described by Sancho et al. (2007). Inside the chamber is a turntable with eight sample holders, a LED unit for the illumination of one sample with visible light and UV-radiation, and two humidity sensors close to the samples (approximately 1 cm) calibrated for the Martian atmosphere, each equipped with a Pt100-sensor. The gas flow through the EC is generated by a gas mixing system, which can control up to five gases and EC humidification. The pressure inside is controlled by a membrane vacuum pump. The radiation dose is measured with an X92-optometer and a RCH-106-4 probe (Gigahertz-Optik GmbH, Germany) at wavelengths ranging from 250 to 400 nm.

### Experimental conditions

Over the complete experimental time course, the EC had a continuous gas flow of 20 L h^−1^ (at 101,325 Pa). The gas consisted of 95% CO_2_ and 5% air (4% N_2_, 1% O_2_). The pressure inside the EC was approximately 750 Pa, the temperature varied diurnally between −50°C (night) and 20°C (day), whereas the relative humidity (with respect to ice) ranged between approximately 75% (night) and near 0% (day). Table 1 summarizes the experimental conditions used in the chamber in comparison with environmental conditions on the surface of Mars.

**Table 1:** Experimental conditions used in the Mars Simulation Facility (MSF) of the DLR Institute of Planetary Research in Berlin in comparison to Mars surface conditions.

**Ranges of experimental and environmental parameters**	**Mars-like conditions in the lab**	**Mars conditions**
Relative humidity	0.1–75%	0–100% (at soil near saturation according to Harri et al., 2014)
Pressure	750 Pa	680–790 Pa (Hassler et al., 2013)
Temperature	(night −50°C to day +20°C), simulation of equatorial to mid latitude regions	Mean value (−55°C, −130°C at the poles to +27°C at the equatorial regions)
Atmospheric gas composition	CO_2_ (95%), N_2_ (4%), O_2_ (0.15%)	CO_2_ (95.97%), N_2_ (1.89%), Argon (1.93%), O_2_ (0.15%), (de Vera et al., 2014)
Radiation	Xe-UV (2,200 to 2,200 nm)	Solar radiation (>200 nm) (Schuerger et al., 2003; de Vera et al., 2014)

**Figure 3:**
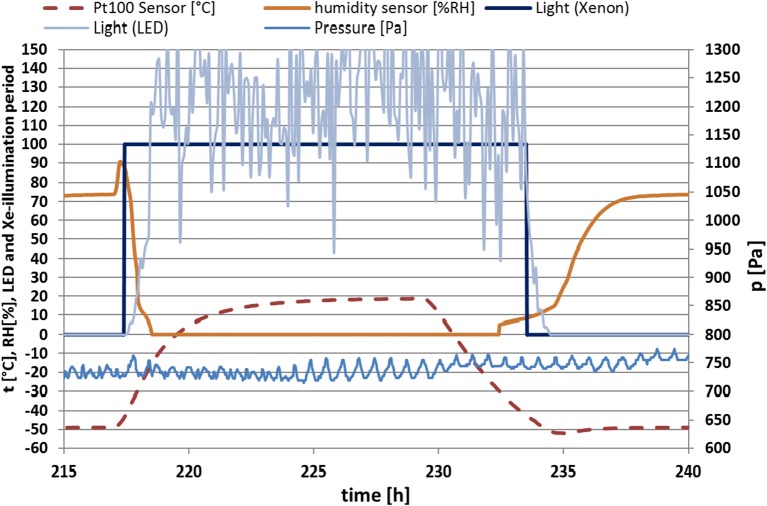
Mars-like environmental conditions [temperature (*t*), relative humidity (RH), illumination period (by LED and Xenon) and pressure (*p*)] of a selected exemplary temporal section extracted from the 30 day data set of the Mars simulation experiment.

Four samples of *C. gyrosa* were illuminated with xenon light (full UV-radiation), and four samples were exposed to niche-led conditions. The LED lamp produces photosynthetic active radiation (PAR) and is used in addition to scattered UV-radiation produced by the xenon lamp. This allowed us to approach as closely as possible the conditions that could be faced in micro-fissures and micro-caves (so-called micro-niches) in rocks and the Martian soil. In these fissures and caves, only a short exposure period of scattered or direct light for a few minutes depending on the angle of the sun could be measured (Schuerger et al., 2003; de Vera et al., 2014). The LED unit was active for 16 h and switched off 8 h daily to simulate the Sun's diurnal cycle. During the same time period, the xenon-lamp was switched on and off with the exception that on weekends the UV-lamp remained off because only manual operation was possible. The resulting average radiation doses were 5.19 KJ m^−2^ for niche-led conditions (samples 1–4) and 127.52 KJ m^−2^ for full UV-radiation (samples 5–8). Figure 3 shows the environmental conditions.

### Chlorophyll *a* fluorescence analysis

To assess photosynthetic activity of the lichen photobiont after a 4-week exposure to simulated Mars conditions, the maximum quantum yield (QY) calculated as Fv/Fm (Schreiber et al., 1994), of the photosystem II (PS II) was measured in pre-activated samples by chlorophyll *a* fluorescence analysis. Reactivation of samples was performed for 72 h in a growth chamber at 10 °C and 100 μmol m^−2^ s^−1^ photosynthetic photon flux density (PPFD) for a daily 12 h photoperiod and moistened twice a day with mineral water. These conditions mimicked the environmental conditions accompanying high water availability corresponding to physiological activity such as early morning dew formation, low temperatures, and low light conditions (Lange et al., 1970). Chlorophyll *a* fluorescence of re-activated samples was measured with a photosynthesis yield analyzer (Mini-PAM, Walz GmbH, Germany) as described by Sancho et al. (2007).

### Epifluorescence and confocal laser scanning microscopy

Changes in cell viability and metabolic activity of *C*. *gyrosa* exposed to simulated Mars conditions were assessed by epifluorescence and confocal laser scanning microscopy (CLSM). Before observation, the lichen samples were rehydrated with mineral water and reactivated for 12 h at 10°C. Subsequently, samples were incubated for an additional period of 24 h in a Sanyo MLR-351 plant growth chamber under environmental parameters consisting of 21°C, 60–70% relative humidity, and 12 h dark/12 h illumination with 250 μmol m^−2^ s^−1^ photosynthetically active radiation. Thin sections (80–100 μm) of re-activated lichen samples were prepared with a vibratome and immediately visualized using a Zeiss Apotome epifluorescence microscope (Zeiss, Hamburg, Germany) with a HXP 120 lamp and an Axiocam 506 digital camera. The cube filters for epifluorescence were 38 HE GFP (excitation 450–490 nm and emission 500–550 nm) and cube 50 Cy5 (excitation 625–655 nm and emission 665–715 nm).

Thin sections from re-activated thallus samples for CLSM were stained with 25 μM of the fluorescent dye FUN-1® (Molecular Probes, Oregon, USA) in 2% glucose and 10 mM HEPES buffer (pH 7.0) and incubated at 25°C for 3 h. FUN-stained samples were then observed using a Zeiss LSM 7 DUO Confocal Microscope (Zeiss GmbH, Germany) with a 10X EC Plan-Neofluar objective. Images were acquired at eight bits with 1,024 × 1,024 pixels format using the 488 nm line of an argon ML laser as the only excitation source and a mean beam splitter of 488/561. Emission bands for each channel were 505–550 nm (for green), 581–620 nm (for red), and 672–758 nm (for blue). Image stacks were taken through the z axis at 4 μm intervals through the whole section and analyzed with ZEN 2011 software (Zeiss GmbH, Germany). Images were obtained by combining the three fluorescence signals.

### Field emission scanning electron microscopy

To accurately assess thallus anatomy and morphological changes in *C*. *gyrosa* samples exposed to simulated Mars conditions, field emission scanning electron microscopy (FESEM) with energy dispersive X-ray spectroscopy (EDS) was conducted. Before FESEM examinations, thin sections of the reactivated thallus samples were fixed with 2.5% glutaraldehyde in 0.1 M cacodylate-buffer (pH 7.4) at 4°C for 2 h, washed three times in cacodylate-buffer for 5 min per wash period, and post-fixed in 1% osmium tetroxide for 1 h at 4°C. The samples were then dehydrated by subsequent serial dilutions in ethanol and acetone and then dried in a critical point drying device (Leica EM CPD300) at 34.5°C. Finally, the fixed lichen samples were sputter-coated with a thin gold film and observed with an FEI Teneo FESEM (FEI Company, Eindhoven, The Netherlands) using the secondary electron detection mode with an acceleration voltage of 5 kV for ultra-high resolution images and 10 kV for elemental microanalysis and mapping.

### DNA-based analyses

Polymerase chain reaction (PCR) analyses were performed to verify deoxyribonucleic acid (DNA) damage by both random amplification of polymorphic DNA (RAPD) fingerprinting and single gene amplifications. DNA was extracted from rehydrated lichen samples using the Nucleospin Plant kit (Macherey-Nagel, Düren, Germany) following the protocol optimized for fungi. Amplification of the internal transcribed spacer (ITS) regions and the large and small subunits ribosomal RNA genes were performed using BioMix (BioLine GmbH, Luckenwalde, Germany) after adding 5 pmol of each primer and 20 ng of template DNA at final volume of 25 μl. The amplification was carried out using a MyCycler Thermal Cycler (Bio-Rad Laboratories GmbH, Munich, Germany) equipped with a heated lid. Conditions for rDNA regions amplification and primer sequences are reported in Tables 2, 3, respectively. For the mycobiont, the RAPD protocol of Selbmann et al. (2011) was applied. For RAPD of the *C*. *gyrosa* photobiont, the OPA 13 primer (Table 3) was used and conditions for amplification included several steps: (i) a denaturation step at 94°C for 4 min; (ii) denaturation at 96°C for 30 s; (iii) annealing at 49°C for 60 s; and (iv) extension at 72°C for 30 s. The final three steps were repeated 40 times with a final extension at 72°C for 6 min. Band intensity was measured and compared using Image J software, version 1.45 (Schneider et al., 2012).

**Table 2:** Amplification conditions.

**Samples**	**DNA region**	**Primers**	**First denaturation**	**Denaturation**	**Annealing**	**Extension**	**Final extension**
					**35 cycles**		
Mycobiont	ITS (700 bp)	ITS5-ITS4	95°C for 2 min	95°C for 30 s	55°C for 30 s	72°C for 30 s	72°C per 5 min
Mycobiont	LSU (1,200 bp)	ITS5-LR5	95°C for 3 min	95°C for 45 s	52°C for 30 s	72°C for 3 min	72°C per 7 min
Mycobiont	LSU (2,000 bp)	ITS5-LR7	95°C for 3 min	95°C for 45 s	52°C for 30 s	72°C for 3 min	72°C per 7 min
Photobiont	SSU (1,000 bp)	NS1-NS2	94°C for 3 min	94°C for 45 s	55°C for 1 min	72°C for 3 min	72°C for 5 min
Photobiont	SSU (2,000 bp)	NS1-NS4	94°C for 3 min	94°C for 45 s	55°C for 1 min	72°C for 3 min	72°C for 5 min
Photobiont	SSU (3,000 bp)	NS1-18L	94°C for 3 min	94°C for 45 s	55°C for 1 min	72°C for 3 min	72°C for 5 min

**Table 3:** Primer sequences.

**Primer**	**Sequence (5′ -> 3′)**	**References**
ITS4	TCCTCCGCTTATTGATATGC	White et al., 1990
ITS5	GGAAGTAAAAGTCGTAACAAGG	White et al., 1990
LR5	TCCTGAGGGAAACTTCG	Vilgalys and Hester, 1990
LR7	TACTACCACCAAGATCT	Vilgalys and Hester, 1990
NS1	GTAGTCATATGCTTGTCTC	White et al., 1990
NS2	GGCTGCTGGCACCAGACTTGC	White et al., 1990
NS4	CTTCCGTCAATTCCTTTAAG	White et al., 1990
18L	CACCYACGGAAACCTTGTTACGACTT	Hamby et al., 1988
(GGA)_7_	GGA GGA GGA GGA GGA GGA GGA	Kong et al., 2000
OPA 13	CAGCACCCAC	Ho et al., 1995

### Pyrolysis-gas chromatography/mass spectrometry (Py-GC/MS)

Chemical analyses of lichen thalli were carried out by direct pyrolysis-gas chromatography/mass spectrometry (Py-GC/MS) using a double-shot pyrolyser (Frontier Laboratories, model 2020i) attached to a GC/MS Agilent 6890N as described in Miller et al. (2016). One milligram each of *C. gyrosa* samples from the control, niche-led conditions, and exposed-to-full UV sets were placed in small crucible capsules and introduced into a pre-heated micro-furnace at 500°C for 1 min. The volatile pyrolysates were then directly injected into the GC/MS for analysis. The gas chromatograph was equipped with a HP-5ms-UI, low polar-fused silica (5%-phenyl-methylpolysiloxane) capillary column of 30 m × 250 μm × 0.25 μm film thickness. The oven temperature was held at 50°C for 1 min and then increased to 100°C at 30°C min^−1^, from 100 to 300°C at 10°C min^−1^, and then stabilized at 300°C for 10 min. The carrier gas was helium at a controlled flow of 1 ml min^−1^. The detector was an Agilent 5973 mass selective detector, and mass spectra were acquired at 240°C under electron impact (70 eV of energy). Masses were scanned from m/z 40–600. Each sample was pyrolyzed twice without perceptible differences between the chromatograms of the same sample. Compound assignment was achieved via comparison with already published data or stored in digital libraries (NIST and Wiley libraries).

## Results and discussion

### Mars-like conditions

Before examining and analyzing the results, it has to be clarified that Mars-like conditions in reference to our present work means that we cannot simulate the reduced Martian gravity and direct exposure to heavy radiation particles in the laboratory; these conditions are expected to happen on the surface of Mars because of the lack of a protective global magnetic field; however, by simulating Mars-like niche conditions we are approaching conditions with reduced radiation (that could be expected in micro-fissures and micro-caves close to the surface of Mars) or reduced sunlight intensity caused by dust in the atmosphere.

### PSII activity during the exposure to simulated mars-like conditions

**Figure 4:**
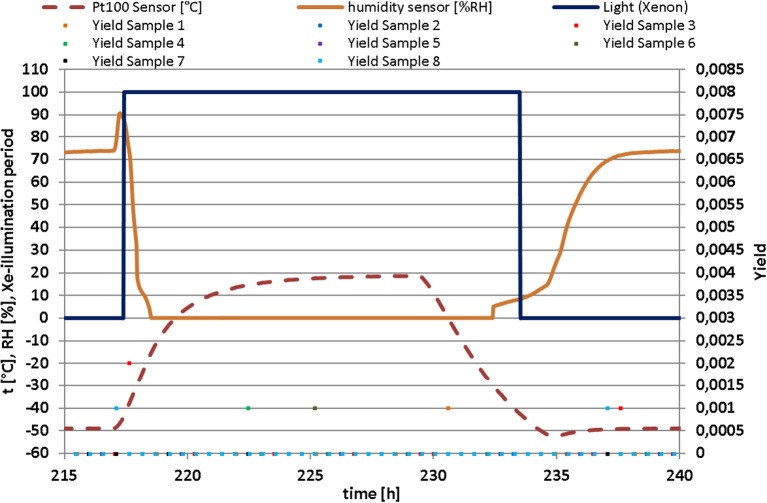
Measurements of photosynthesis activity of eight samples. The photosynthesis activity is shown by the Yield values under Mars-like environmental conditions [temperature (*t*), relative humidity (RH), illumination period by Xenon-lamp] of a selected exemplary temporal section extracted from the 30 day data set of the Mars simulation experiment. No effective photosynthetic activity was measured during the simulation.

Photosynthetic activity measurements were executed in 30 min steps. The yield-value dropped from 0.35 down to approximately 0 for all samples within the first hour of the experiment. Over the remaining experimental period, the photosynthetic activity of all samples ranged from very low to non-existent (Figure 4). Although the photosynthetic light reaction was measured before and after the experiment, it can be concluded that *C. gyrosa* was not photosynthetically active during the Mars exposure experiment. The lichen therefore coped with Mars-like conditions by entering a dormant stage and would not be able to survive on Mars in the long-term in the absence of photosynthetic activity.

### PSII activity before and after the exposure to simulated mars-like condition0073

**Figure 5:**
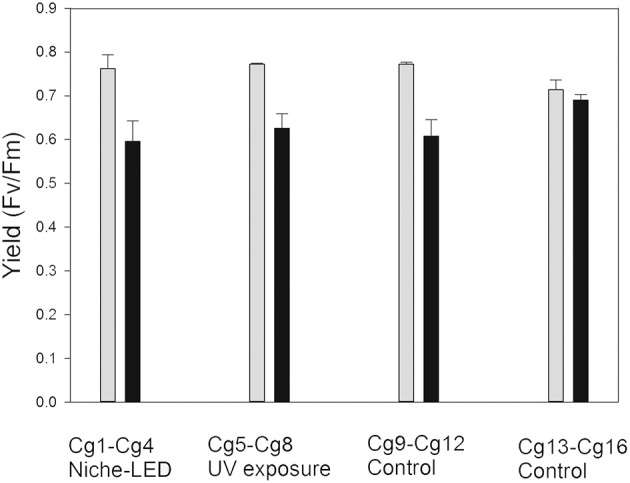
Photosynthetic performance of photosystem II (PSII) by chlorophyll *a* fluorescence: Before exposure (light gray) and after Mars simulation experiment (dark gray).

The PSII was measured as the maximum QY for the different set of samples before exposure to simulated Mars-like conditions and showed a homogeneous tendency without significant differences between them (Figure 5). However, after exposure to the Mars-like environment, the mean values showed differences over a range of 20% for both sample sets, including the control set (Cg9–Cg12, Figure 5). These differences were higher than those of the second control set (Cg13–Cg16) with 0.5%.

These results indicate that a simulated Mars-like environment with attenuated niche-like conditions and full impact of Mars UV radiation (full UV) did not significantly affect the photosynthetic activity of the re-activated *C*. *gyrosa*. In other words, the photosynthetic activity of the lichen thalli recovered efficiently after re-activation (rehydration after exposure to Mars-like conditions).

### Morphology and cell viability after exposure to simulated mars-like conditions

**Figure 6:**
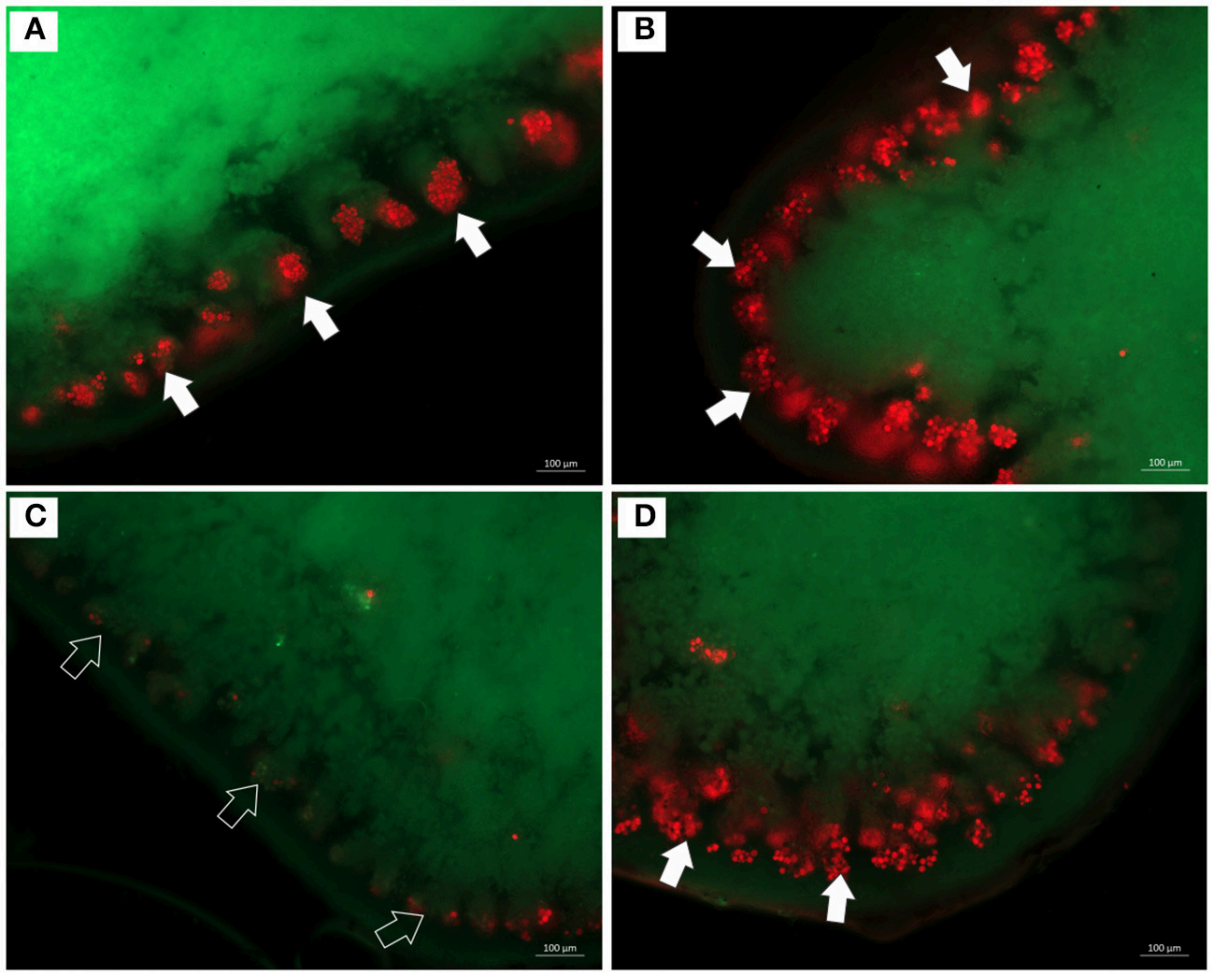
Epifluorescence microscopy images of thin sections of *C*. *gyrosa* lichen thalli before **(A)**, and after exposure to niche-led conditions **(B)**, and to full UV radiation **(C,D)**. Red color represents chlorophyll autofluorescence from algal cells (white arrows). In samples fully exposed to UV radiation **(C,D)**, almost no autofluorescence was observed in zones occupied by algal clusters (black arrows).

Chlorophyll autofluorescence monitored by epifluorescence microscopy for evaluating cell viability of the photobiont showed slight differences in the red light emission (chlorophyll autofluorescence) among the three sets of samples (Figure 6). The *C*. *gyrosa* thalli exposed to niche-led conditions exhibited red fluorescence similar to the control samples (Figures 6A,B). In samples fully exposed to UV radiation, algal cells were less abundant, and almost no autofluorescence was observed in zones occupied by algal clusters (Figure 6C). This indicates an irradiation-induced degradation of chlorophyll or an inefficient reactivation of lichen physiological processes after rehydration. However, minimal red fluorescence was recorded in the algal layer (Figure 6D).

**Figure 7:**
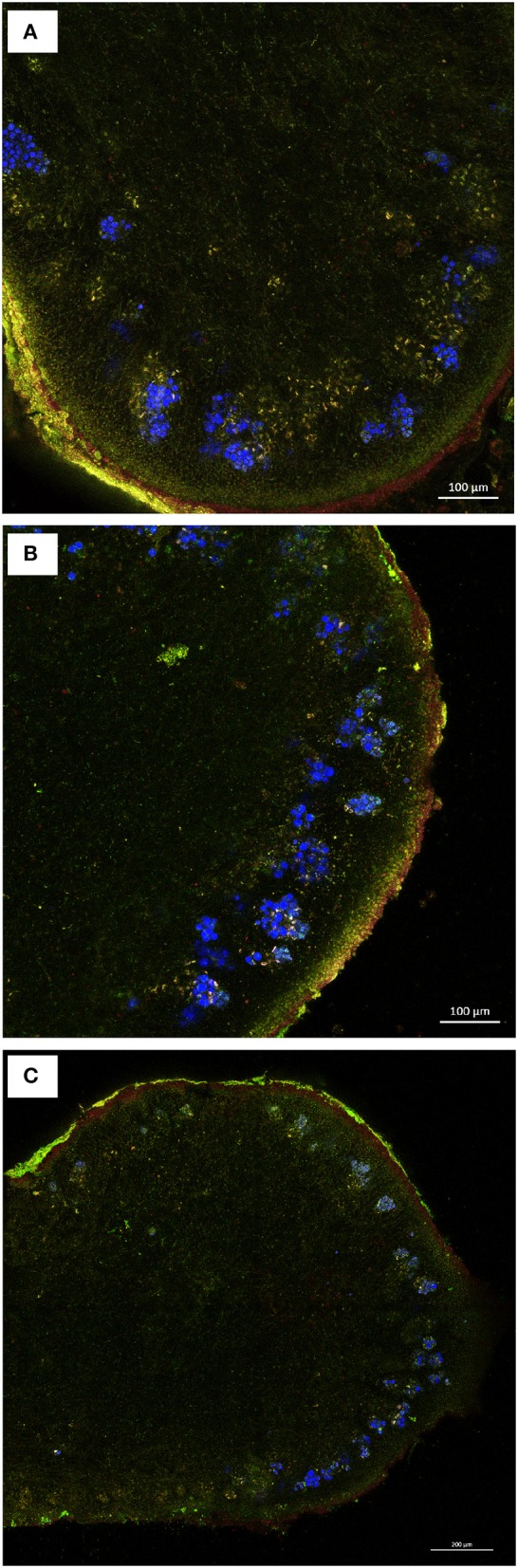
CLSM images of FUN-1 stained thin sections of *C*. *gyrosa* lichen thalli before **(A)**, and after exposure to niche-led conditions **(B)**, and to full UV radiation **(C)**. The FUN-1 stain yielding green fluorescent hyphae with red fluorescent vacuoles represent metabolically active hyphae, which are particularly observed in the proximity of the algal clusters. Blue cells represent chlorophyll autofluorescence. Green fluorescent hyphae without red structures, indicating lack of viability, are observed in the innermost medullary part of the lichen fully exposed to UV radiation **(C)**.

The FUN-1 stain was used for detecting the presence of metabolically active fungal cells by CLSM due to intracellular processing of the vacuolar dye (red fluorescence). Algal cells could also be observed and identified by recording their autofluorescence signal in the blue channel (Figure 7). CLSM analyses of the lichen photobiont were in line with the epifluorescence-monitored chlorophyll autofluorescence. Living algal cells (with intense blue fluorescence) were more abundant in the control (Figure 7A) and samples exposed to niche-led conditions (Figure 7B) than to full UV (Figure 7C). The latter samples showed clear asymmetry of the algal layer in the thin lichen cross-sections as intense chlorophyll autofluorescence was intermixed with faded blue fluorescent cells (Figure 7C). Concerning the mycobiont, similar levels of metabolization of the FUN-1 stain yielding green fluorescent hyphae with clear red fluorescent vacuoles were observed in the control and irradiated samples, particularly in the proximity of the algal layer, suggesting metabolically active fungal cells (Figures 7A,B). Green fluorescent hyphae without red structures were particularly observed in the innermost medullary part of the lichen, pointing to a lack of viability in these regions.

**Figure 8:**
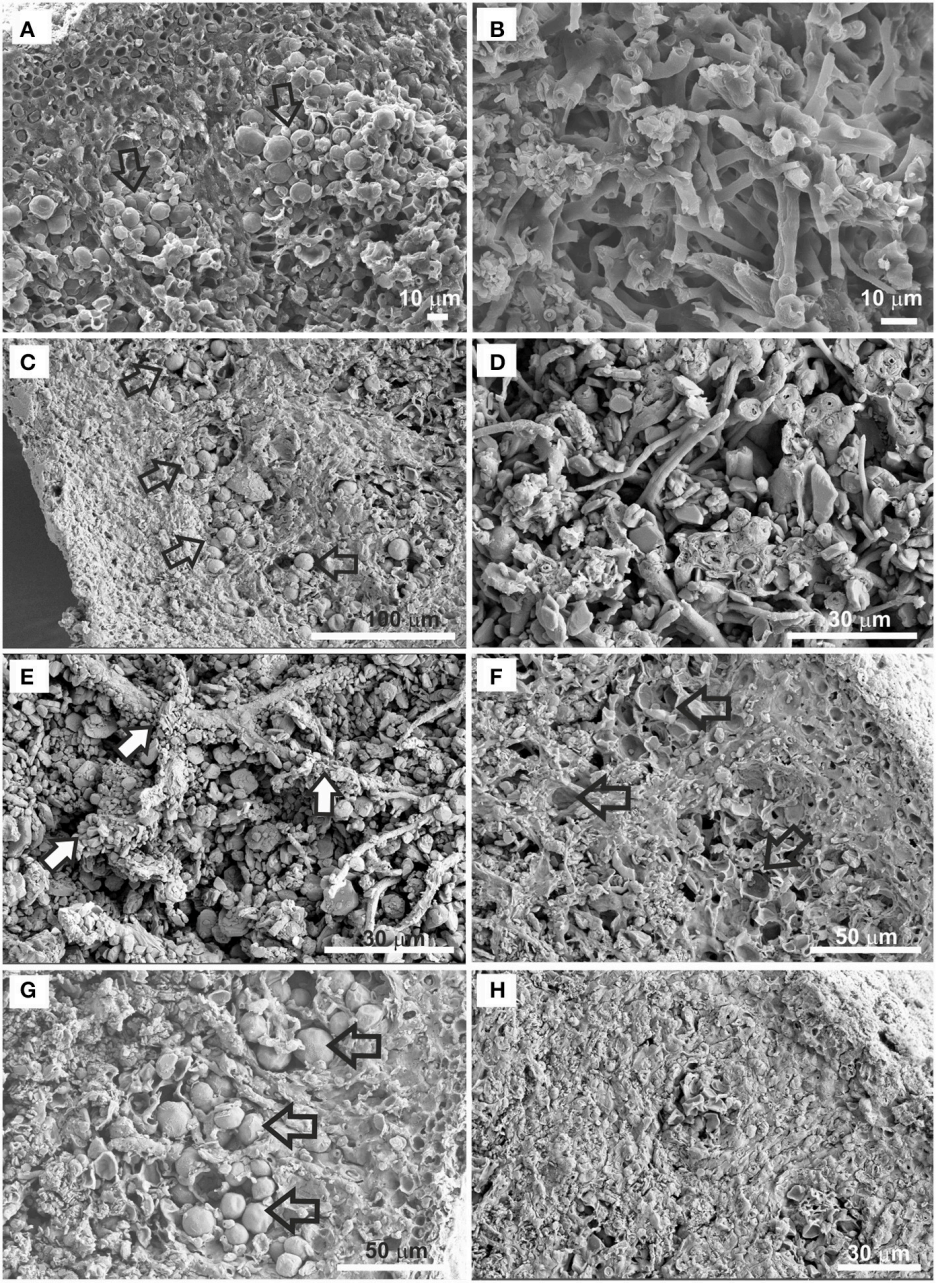
FESEM images of *C. gyrosa* thin sections before **(A,B)**, and after exposure to niche-led conditions **(C–E)**, and to full UV radiation **(F–H)**. The control sample shows photobiont cells arranged in clusters (**A**, arrows), and the medulla with loosely interwoven fungal hyphae **(B)**. The samples exposed to niche-led conditions show good anatomical preservation of the algal clusters (**C**, arrows) and of the lichen medulla **(D)**. Highly mineral-encrusted hyphae (**E**, white arrows) were observed in all samples. Disrupted photobiont cells (**F**, arrows), as well as well-preserved algal cells (**G**, arrows) are observed in the full UV exposed samples. Tightly packed fungal cells are observed for the full UV exposed samples **(H)**.

FESEM of the thin lichen sections used for assessing thallus structural changes and damage to cell walls revealed differences among the three set of samples (Figure 8). The control sample showed the characteristic *C. gyrosa* thallus structure with the algal layer composed of photobiont cells arranged in clusters (Figure 8A) and a medulla with loosely interwoven fungal hyphae (Figure 8B). Both control and niche-led samples showed good anatomical preservation of the algal clusters in addition to the individual cells (Figure 8C). In addition, the medulla did not seem to be structurally affected and consisted of interlaced hyphae with numerous voids (Figure 8D), indicating that the niche-like exposure conditions did not disturb either the lichen thallus structure or the morphology. Interestingly, FESEM examinations revealed highly mineral-encrusted hyphae in all samples, predominantly in the inner medullary structure (Figure 8E). According to crystal shape and EDS analyses, this mineral is consistent with whewellite (calcium oxalate monohydrate), an insoluble organic salt secreted by the cells for several purposes, including calcium regulation, protection, and structural strengthening (Giordani et al., 2003). Whewellite crystals are widespread among organisms and have been reported on stone surfaces that were densely colonized by lichens (Pereira de Oliveira et al., 2011). Extracellularly deposited whewellite-crystals were also observed by Meeßen et al. (2013), Böttger et al. (2014), and De la Torre et al. (2017) on *C*. *gyrosa* medullary hyphae.

On the other hand, the asymmetry observed by CLSM relating to the numbers and distribution of algal cells throughout the lichen thalli of the full UV exposed samples was clearly detected by FESEM. Figure 8F shows part of a lichen thallus with disrupted photobiont cells in the zones occupied by algal clusters, whereas other parts of the same thin section showed well-preserved algal cells (Figure 8G). The loose inner medulla of the niche-led samples (Figure 8C) contrasted with the tightly packed fungal cells of the full UV exposed samples conglutinated with extracellular cementing substances, particularly in the proximity of the algal layer (Figure 8H). Taken together, these data indicate that *C. gyrosa* samples exposed to full UV radiation show a slight delay in physiological activity recovery after rehydration in comparison to the lichen thalli exposed to niche-led conditions. In a review by Holzinger and Karsten (2013) about desiccation tolerance of aeroterrestrial algae, it was reported that green algae rapidly reduced photosynthetic activities during desiccation but recover quickly after rehydration. Similarly, Aubert et al. (2007) showed that samples of the lichen *X. elegans* in high mountain environments instantly recovered respiration after hydration and both photobiont and mycobiont cells did not suffer irreversible desiccation-induced damage.

### DNA integrity evaluation

**Figure 9:**
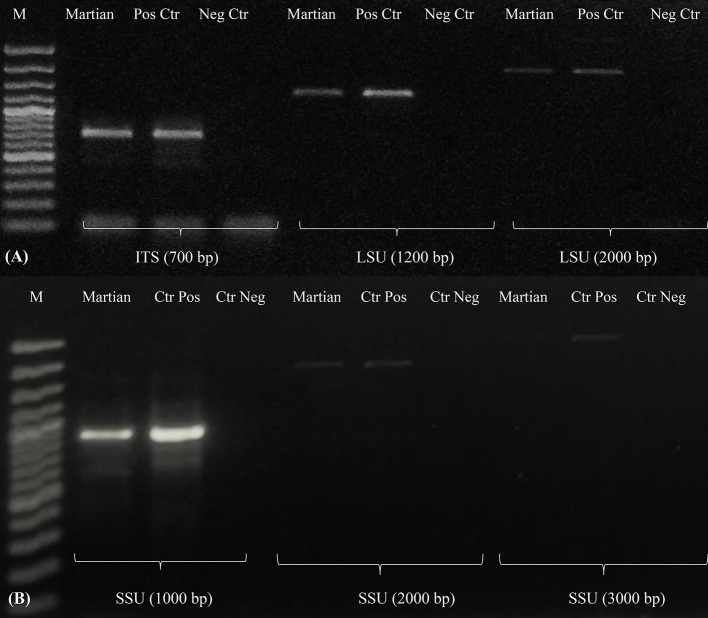
Single gene amplification of *C. gyrosa* mycobiont **(A)** and photobiont **(B)**. Gene length as reported in Table 2. MW, DNA ladder; Martian, Martian conditions; CTR Pos, Untreated Control; CTRL Neg, PCR Negative Control.

DNA integrity was assessed by amplifying three different genes both for the mycobiont and photobiont and also by using a fingerprinting analysis to analyze whole genome integrity. PCR-stop assays are a powerful method for evaluating DNA damage since lesions block the progression of DNA polymerase (Kumar et al., 2004; Trombert et al., 2007), and long amplicons have a higher likelihood of undergoing DNA damage than short PCR amplicons (Rudi et al., 2010). In addition, in genomic PCR fingerprinting that amplifies short genomic DNA portions, PCR band intensity mainly decreased in the highest molecular weight fragments in single-gene PCR (Atienzar et al., 2002; Atienzar and Jha, 2006). Amplicons were obtained both for ITS and large subunit (LSU) regions of the mycobiont (Figure 9A). These approaches have been applied to dried *Cryomyces antarcticus* colonies exposed to UV irradiation (Selbmann et al., 2011) and then successfully applied under both simulated space- and Mars conditions-, ionizing radiation-, and alpha particles-exposed samples of the same fungus (Onofri pers. com.; Pacelli et al., 2017a,b,c). A reduction of amplicon intensity was observed with increasing genetic marker length. Nevertheless, DNA was still intact and perfectly detectable at the highest molecular weight band (2,000 bases). Single gene amplifications of photobiont DNA (Figure 9B) revealed that DNA was amplified at both 1,000 and 2,000 bases. We could not obtain a PCR product (band) for the longest length tested (3,000 bases).

**Figure 10:**
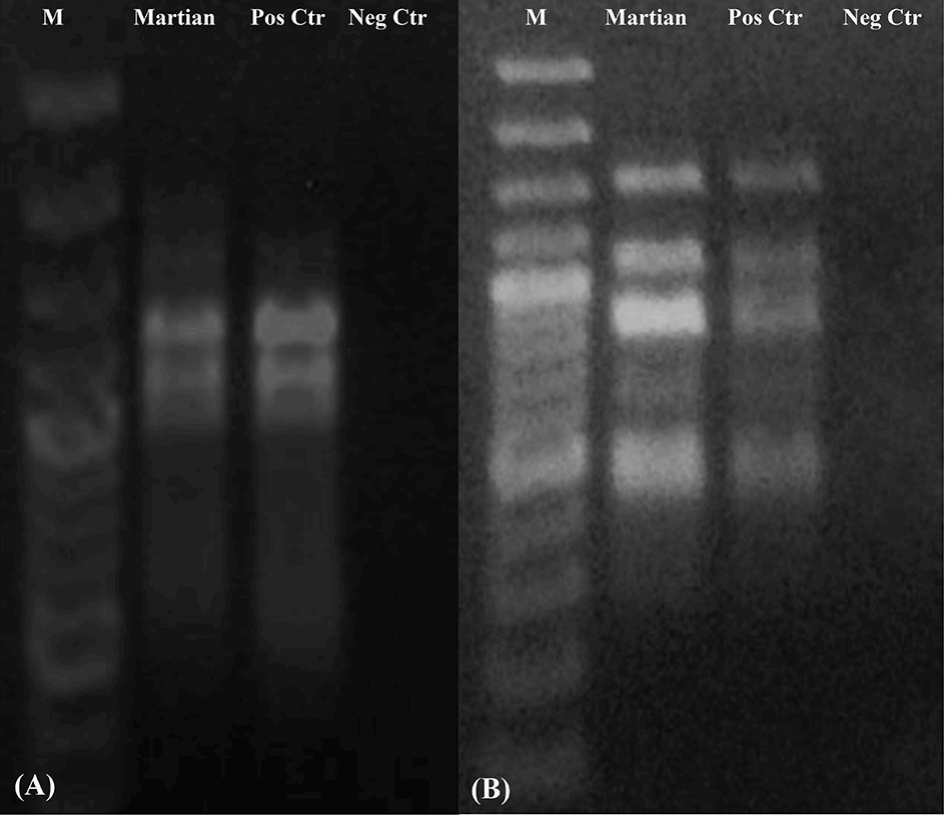
RAPD profile of *C. gyrosa* mycobiont **(A)** and photobiont **(B)**. Samples correspondence as reported in Figure 9.

RAPD profiles of samples under Mars-like conditions were identical to those of both the mycobiont and photobiont since no band disappearance was observed; the whole genome was conserved (Figure 10). For DNA analyses, the photobiont was the most affected by Martian conditions, showing a reduction up to 50% for the maximum gene length (Figure 10B), while the mycobiont DNA was perfectly detectable at every examined gene length (Figure 10A). Notably, in our experiments DNA could be easily extracted and detected even after being exposed to Mars-like conditions. The low temperatures in space was shown to reduce DNA breaks by half compared to ambient Earth temperatures (Lindahl, 1993), and the dry space environment was shown to preserve DNA (Lyon et al., 2010). Furthermore, it has been already reported that the low temperatures and dry conditions on Mars may preserve DNA in a better way in the long-term than do the conditions on Earth (Kanavarioti and Mancinelli, 1990; Sephton, 2010). Other studies on *C. antarcticus* DNA damage after exposure to the Martian atmosphere similarly demonstrated that fungal DNA was not affected and remained perfectly detectable by PCR (Pacelli et al., 2017b).

### Analytical pyrolysis (Py-GC/MS)

**Figure 11:**
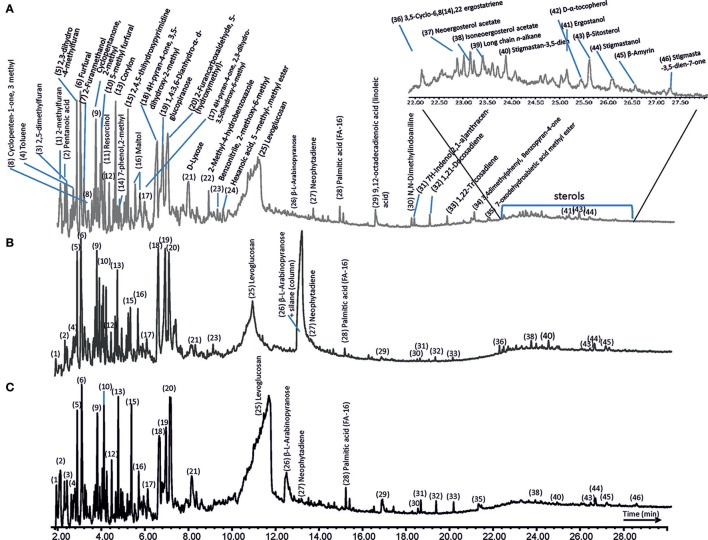
Analytical pyrolysis (Py-GC/MS) at 500°C of the *C*. *gyrosa* lichen thalli before **(A)**, and after exposure to niche-led conditions **(B)**, and to full UV radiation **(C)**.

The GC/MS total ion chromatograms (TIC) of the pyrolysates of the three sets of samples are shown in Figure 11. They were very similar and consisted mainly of furanes, furfural, and methyl derivatives of these compounds. They were polysaccharide-derived xyloses and hexoses which constituted about 95% of the lichen molecular composition according to Schellekens et al. (2009). These compounds were also found by analytical pyrolysis to be common products of metabolic lichen thalli activities (Saiz-Jimenez et al., 1991). The most abundant compound of the three TICs was levoglucosan (peak 25, Figure 11), which is a six carbon ring structure formed from the carbohydrate (such as starch or cellulose) pyrolysis. One of the differences observed in the chromatograms was the presence of L-arabinopyranose in the full UV-irradiated lichens (peak 26, Figure 11C). This was a furan-related aldopentose, which is found in nature as a component of biopolymers such as hemicellulose. Neophytadiene, a marker of chlorophyll in algae and cyanobacteria (Saiz-Jimenez et al., 1990), was present in all of the samples (peak 27, Figure 11). Its relative abundance decreased in the fully UV-irradiated lichens (Figure 11C); nevertheless, in the latter case, the peak was partially masked by L-arabinopyranose (peak 26).

A series of sterols and terpenes were identified in all the TICs (from min 22 to 28). Our results revealed a mixture of C_28−30_ sterols (peaks 37–41 and 43–46, Figure 11), which included ergosterol acetates, ergostanol, stigmastan-3,5-dien, and β-sitosterol. They were plant cell membrane components and were lichen secondary metabolites, which have been widely studied due to their medical and pharmaceutical applications. Ergosterol is regarded as the typical membrane constituent of fungi and serves as a bioregulator of membrane fluidity, asymmetry, and integrity. Sufficient ergosterol content is necessary for fungal cell growth and normal membrane function. The TIC of the niche-led samples showed a relative increase in the presence of sterols (Figure 11B), while a clear decrease is observed for the full UV-irradiated samples (Figure 11C). Taking into account that the fluidity of biological membranes is influenced by the amount of sterols (Russel, 1989), this result could indicate surface damage to the membrane resulting from high UV radiation doses. Furthermore, the decrease in the relative abundance of β-sitosterol of the full UV-irradiated samples (Figure 11C) could be due to its dehydration and subsequent transformation into stigmasta-3,5-diene (León-Camacho et al., 2004). In line with the previous findings, D-α-Tocopherol (vitamin E; Peak 42), a key lipid-soluble antioxidant in membranes (Munne-Bosch and Alegre, 2002), was found solely in the control sample (Figure 11A). Fatty acids (FAs) are homologous and typically found in the molecular composition of lichens. Nevertheless, only pentanoic, palmitic, and linoleic acids are detected in the TICs of the lichens (peaks 2, 28, and 29, respectively). The low FA abundance was due to the incompatibility with the apolar columns used in GC and to secondary reactions that occur at the elevated temperatures applied during the pyrolysis (Dignac et al., 2006). A few nitrogen (N)-containing compounds were detected among the pyrolisates (peaks 23, 30, and 34; Figure 11), which were probably derived from peptides. Their decline in the full UV-irradiated lichens could indicate the use of N-precursors to sustain the synthesis of N-containing compounds under stress conditions. These results show that analytical pyrolysis is a valuable tool for assessing alterations in lichens' chemical composition. For instance, specific compounds could be used as biomarkers of cell membrane damage. There are several main advantages of analytical pyrolysis use in for astrobiology: (i) easy sample preparation (drying and milling); (ii) fast analysis time; (iii) very high reproducibility; and (iv) only small amount of sample is required (0.5–5 mg).

## Conclusions

Our multidisciplinary approach allowed us to assess the viability, structure, DNA integrity, and chemical composition of the lichen *C. gyrosa* after exposure to simulated Mars conditions. Although the lichen samples did not have the capability to be photosynthetically active during the 30-day exposure to the Mars-like environment, their photosynthetic activity efficiently recovered after re-activation of the lichen thalli. However, microscopic examination revealed a slight delay in the recovery of *C. gyrosa* after exposure to full UV conditions in comparison to the lichen thalli exposed to niche-led conditions. Good anatomical preservation of the algal clusters and fungal hyphae from the medulla indicated that the niche-led exposure conditions did not disturb either lichen thalli structure or morphology. No DNA damage was observed in the fungal component, while the amplicon for the algal component disappeared after exposure to simulated Mars conditions, suggesting that the photobiont is the most affected component after exposure to simulated conditions. Analytical pyrolysis revealed a similar composition for the UV- and non-irradiated lichens, which were dominated by the typical cell wall constituents. Nevertheless, the changes in the relative abundance of specific sterols in the lichens exposed to full UV conditions indicate that the UV irradiation induced injury to the lichen membranes.

We showed by experimental means that the surface of Mars would be harmful for the tested lichen *C. gyrosa*; therefore, the surface of Mars would not be a habitable place for *C*. *gyrosa*. In contrast, niches on Mars close to the surface, in which the organism is protected against UV-radiation, might be retreat areas for this lichen. It means that we have to clearly differentiate between the Martian surface (not habitable), the subsurface (potentially habitable), and niches near the surface (partially habitable in fissures, cracks, and micro-caves in rocks, de Vera et al., 2014).

## Author contributions

RT: coordinated the complete study, and determined the chlorophyll a-fluorescence. AM: provided several analysis-methods: CLMS (Confocal-Laser-Scanning Microscopy), Epifluorescence and FESEM/Field Emission Scanning Microscopy), and TEM (Transmission Electron Microscopy). JR: provided the analytical pyrolysis (Py-GC/MS) methodology. CP: provided the DNA integrity evaluation (RAPD and PCR methods). SO: coordinated and supervised the DNA integrity evaluation (RAPD and PCR methods). LG: selected the lichen sample (field campaigns) and supervised the writing of the manuscript. BC: provided the CLMS analysis. AL: provided the technical control of the Mars Simulation Chamber at DLR-Berlin. DW: prepared the biological samples for the simulation test and evaluated the results. JV: coordinated and supervised the Mars simulation test.

### Conflict of interest statement

The authors declare that the research was conducted in the absence of any commercial or financial relationships that could be construed as a potential conflict of interest.
